# Effect of Neuroglobin Genetically Modified Bone Marrow Mesenchymal Stem Cells Transplantation on Spinal Cord Injury in Rabbits

**DOI:** 10.1371/journal.pone.0063444

**Published:** 2013-05-02

**Authors:** Wen-Ping Lin, Xuan-Wei Chen, Li-Qun Zhang, Chao-Yang Wu, Zi-Da Huang, Jian-Hua Lin

**Affiliations:** 1 Department of Orthopedic Surgery, The Second Affiliated Hospital, Fujian Medical University, Quanzhou, Fujian Province, People's Republic of China; 2 Department of Orthopedic Surgery, The First Affiliated Hospital, Fujian Medical University, Fuzhou, Fujian Province, People's Republic of China; University of Medicine and Dentistry of New Jersey, United States of America

## Abstract

**Objective:**

This study aims to investigate the potentially protective effect of neuroglobin (Ngb) gene-modified bone marrow mesenchymal stem cells (BMSCs) on traumatic spinal cord injury (SCI) in rabbits.

**Methods:**

A lentiviral vector containing an Ngb gene was constructed and used to deliver Ngb to BMSCs. Ngb gene-modified BMSCs were then injected at the SCI sites 24 hours after SCI. The motor functions of the rabbits were evaluated by the Basso–Beattie–Bresnahan rating scale. Fluorescence microscopy, quantitative real-time PCRs, Western blots, malondialdehyde (MDA) tests, and terminal deoxynucleotidyltransferase-mediated UTP end labeling assays were also performed.

**Results:**

Ngb expression in the Ngb-BMSC group increased significantly. A more significant functional improvement was observed in the Ngb-BMSC group compared with those in the other groups. Traumatic SCI seemingly led to an increase in MDA level and number of apoptotic cells, which can be prevented by Ngb-BMSC treatment.

**Conclusion:**

This study demonstrates that Ngb gene-modified BMSCs can strengthen the therapeutic benefits of BMSCs in reducing secondary damage and improving the neurological outcome after traumatic SCI. Therefore, the combined strategy of BMSC transplantation and Ngb gene therapy can be used to treat traumatic SCI.

## Introduction

Traumatic spinal cord injury (SCI) is one of the most devastating forms of trauma cases. SCI can cause severe functional impairment, paraplegia, and tetraplegia. The pathophysiology of SCI remains poorly understood; however, studies suggest the presence of primary and secondary injury mechanisms. Primary injury results from mechanical damage to the spinal cord that causes spinal cord edema and immediate neuronal death, which is inevitable. After the occurrence of primary injury, the spinal cord undergoes a number of sequential pathologic changes, including hypoxia, reactive oxygen species (ROS) production, lipid peroxidation, ischemia, apoptosis, and inflammation, among others [1], [2]. These secondary injury processes are potential targets for therapeutic intervention [3], [4]. Despite the use of therapeutic agents to protect the injured spinal cord from these secondary pathological processes [5]–[8], no effective treatment for SCI has thus far been developed.

Cell-based gene therapy is a potentially effective approach to traumatic SCI treatment [9]. Among various types of candidate cells, the bone marrow mesenchymal stem cells (BMSCs) exhibit potential because they are readily available and have no ethical issues associated with the transplantation of other types of stem cells such as embryonic stem cells [10] or neural stem cells [11]. BMSCs provide an interesting model for examining the multi-lineage and differentiation potential of stem cells [12]. More evidence shows that BMSC transplantation promotes recovery in SCI [13], [14].

Neuroglobin (Ngb) is a vertebrate globin expressed in the central and the peripheral nervous systems [15]–[17]. Several studies indicate that Ngb overexpression can protect neurons from hypoxia [18] and ischemia [19], [20] by enhancing either hypoxia sensing or hypoxia response. Ngb may scavenge nitric oxide or regulate ROS [21]–[23] as well as provide an unidentified method of apoptosis regulation in neurons [24]. The neuroprotective role of Ngb has been demonstrated both *in vitro* and *in vivo* [25].

On the basis of previous studies, we hypothesized that Ngb upregulation could strengthen the neuroprotective effect of BMSCs. The present study aimed to determine whether Ngb-modified BMSCs could improve the protective effect of BMSC therapy for SCI.

## Materials and Methods

### Isolation, culture, and identification of BMSCs

The use of animals in this study was approved by the Animal Care and Use Committee of the Fujian Medical University. All procedures were in accordance with the guidelines of the National Institutes of Health Guide for the Care and Use of Laboratory Animals (NIH Pub. No. 85–23, revised 1996).

Primary BMSC cultures were set up from New Zealand white rabbits (6 weeks old). The BMSCs were isolated as described previously [26]. Briefly, 6 ml of bone marrow was harvested from the ilium of a rabbit and diluted with the same volume of phosphate-buffered saline (PBS) containing 5000 U of heparin. The mixture was separated by density centrifugation using a lymphocyte separation solution (1.073 g/ml; Sigma – Aldrich, USA) at 1000×g for 15 min at room temperature. The mononuclear fraction interphase was collected and washed twice in PBS. The final pellet was resuspended in 3 ml of Dulbecco's modified Eagle's medium (Gibco, USA) supplemented with 15% fetal bovine serum (Gibco, USA) and penicillin/streptomycin (100 U/ml; Gibco, USA), seeded in a 25 cm^2^ culture flask, and cultured at 37°C under a 5% CO_2_ atmosphere. The non-adherent cells were then discarded after 48 hours of culture. At 80% to 90% confluence, the cells were harvested with trypsin (0.25%) – ethylenediaminetetraacetic acid (Gibco, USA) and replated by splitting, usually 1:3 at a density of 50% to 60%. The BMSCs in passage 3 were used for the experiments.

At passage 3, the BMSCs were trypsinized into single-cell suspensions and stained with fluorescein isothiocyanate-labeled antibodies for flow cytometric analysis, including anti-rabbit CD29, CD34, CD45, and CD90 (BD PharMingen, USA).

### Production of lentivirus and infection of BMSCs

The rabbit *Ngb* gene (Ref sequence: NM_001082133) was synthesized by Sangon Bioengineering Technology and Services Co., Ltd. (Shanghai, China) and confirmed by sequencing. The expression vector pGCFU containing the enhanced green fluorescent protein (*eGFP*) gene was double cut with *AgeI.* An *Ngb* fragment was aligned to produce pGCFU-Ngb. A three-plasmid-envelope system was used to create the lentivirus of pGCFU-Ngb in 293T cells. A quantitative real-time PCR (qRT-PCR) test of the eGFP expression revealed that the titers of the Ngb lentivirus (LV-Ngb) ranged from 0.5×10^9^ TU/ml to 1.0×10^9^ TU/ml.

The BMSCs (1×10^5^) in passage 3 were infected with LV-Ngb at a multiplicity of infection of 100. The BMSCs contained both *eGFP* and *Ngb* genes. Ngb-BMSCs were harvested. The *eGFP* or *Ngb* gene expression was detected using a fluorescence microscope (Olympus Corp., Tokyo, Japan) or the Western blot. Ngb-BMSCs were then prepared at 5×10^6^ in 15 µl of saline solution for the transplantation.

### SCI Model

Ninety-six New Zealand white rabbits weighing between 2.5 and 3.0 kg were used for *in vivo* study. The SCI model was established by epidural balloon compression in the rabbits as described previously [27]. All rabbits received intramuscular injections of anesthetics, including ketamine (50 mg/kg of body weight) and xylazine (5 mg/kg of body weight). Midline skin incisions were performed to expose the T9 to T11 spinous processes. Self-retaining retractors were used on the paraspinous muscles. Laminectomies were subsequently performed at the T10 level. Balloon angioplasty catheters (Medtronic-10851631, 2.0 mm×20 mm, USA) filled with normal saline were inserted into the epidural spaces from the T10 level to the T9 level. The balloons were inflated slowly until a 2 atm pressure was reached. After 5 minutes of compression, the balloons were deflated and removed. The muscles and the skins were sutured in separate layers. After 24 hours, the rabbits were injected with penicillin (30,000 U/kg) and received bladder expressions twice daily until their normal functions returned.

### Transplantation of Ngb-BMSCs

Basso–Beattie–Bresnahan (BBB) scores were obtained 24 hours after SCI. The rabbits were randomly assigned to four groups (n = 24 per group): the Control, NS, BMSC, and Ngb-BMSC groups. The rabbits were anesthetized using the same methods described above. A T9 laminectomy was then performed. In the Control group, no media or cells were injected at the SCI site. In the NS group, 15 μ1 of normal saline was injected at each SCI site by using a microsyringe (Hamilton, Reno, NV). In the BMSC group, 15 µl of BMSC suspension containing about 5×10^6^ cells was injected at each SCI site. In the Ngb-BMSC group, 15 µl of Ngb-BMSC suspension containing about 5×10^6^ cells was injected at each SCI site. After removal of the injector, the muscles and the skin were sutured in separate layers.

### Fluorescence imaging

On days 3, 7, 14, and 21 following SCI, the injured spinal cord tissues were removed and cut into 8 μm-thick serial sections. eGFP expression was observed under a fluorescence microscope (Olympus Corp., Tokyo, Japan).

### qRT-PCR analysis of Ngb in injured spinal cords

The spinal cord segments were first homogenized. Total RNAs were then extracted with Trizol reagents (Invitrogen Life Technologies, USA) and quantified by ultraviolet spectroscopy. Reverse transcription was performed in a 20 μl reaction system with 4 μg of total RNAs treated by RNase-free DNase I (Takara Bio, Inc., Japan) according to the manufacturer's instructions. The PCR primers purchased from Takara Bio, Inc. include the following: GAPDH forward primer, CCACTTTGTGAAGCTCATTTCCT; GAPDH reverse primer, TCGTCCTCCTCTGGTGCTCT; Ngb forward primer: CTGGACCACATCAGGAAGGT; and Ngb-reverse primer, CCCAGACACTTCTCCAGCAT. qRT-PCR was performed using the Thermal Cycler Dice™ Real Time System (TP800, Takara Bio, Inc., Japan). The reaction was conducted in a 20 µl of mixture containing 10 µl of SYBR Premix Ex Taq^TM^ (Takara. Bio, Inc., Japan), 5 µM of each primer, and 1 µl of the DNA extract. The cycling conditions were as follows: an initial cycle of heating at 95°C for 15 seconds, followed by 40 cycles at 95°C for 5 seconds and 40 cycles at 60°C for 30 seconds, with the data acquired at the annealing and extension cycles. After the PCR reaction, a melting curve was constructed at 95°C for 1 minute and 55°C for 1 minute. The temperature was then increased from 55°C to 95°C. Each sample was processed in triplicate. Gene expression was quantified based on the cycle threshold (Ct) value of each sample. The relative quantity of the Ngb mRNA expression was calculated by delta-delta Ct calculation as 2^−[(treated sample delta Ct) – (control sample delta Ct)]^ [28]. The identity of the PCR products was confirmed by DNA sequence analysis.

### Western blot analysis of Ngb in injured spinal cords

The protein homogenates of the spinal cord samples were prepared by rapid homogenization in a lysis buffer (50 mM Tris-Cl, pH  = 6.8; 100 mM dithiothreitol; 2% sodium dodecyl sulfate (SDS); 12% glycerol). The samples were centrifuged at 17,000×g for 15 minutes at 4°C. Protein concentration was determined by Bio-Rad protein assay. The protein lysates (20 µg) were separated on 10% SDS gels and then transferred to polyvinylidene fluoride (PVDF) membranes. After incubation with goat anti-Ngb (1:200; Santa Cruz Biotechnology, USA), the blots were washed with PBS and incubated with mouse anti-goat IgG (1:3,000; Santa Cruz Biotechnology, USA) conjugated with horseradish peroxidase (Santa Cruz Biotechnology, USA). The immunoreactive complexes were visualized by enhanced chemiluminescence and exposed to X-ray films. Protein signals were quantified by scanning densitometry using AlphaEaseFC^TM^ (AlphaInnotech, CA, USA). The results from each experimental group were expressed as the relative integrated intensity compared with beta-actin.

### Neurological evaluation

The hindlimb locomotor function was assessed 1, 3, 7, 14, and 21 days following SCI by using the BBB locomotor test developed by Basso *et al*. [29]. The hindlimb movements during locomotion were quantified using a scale ranging from 0 to 21, where 0 reflects no locomotor activity and 21 reflects normal performances. The rabbits were placed in a 2 m×3 m open field and observed for 5 minutes at each time point by two observers who were blinded to the experimental protocol. The observers gave their scores independently.

### Malondialdehyde (MDA) levels in injured spinal cords

The extent of lipid peroxidation in the injured spinal cords was estimated by the malondialdehyde (MDA) level, which was measured using the commercial assay kits produced by Nanjing Jiancheng Bioengineering, Nanjing, China. The MDA level in the injured spinal cords were determined by the thiobarbituric acid method [30] and expressed in nmol/mg proteins.

### Terminal deoxynucleotidyltransferase-mediated UTP nick end labeling (TUNEL) assays

The TUNEL assays were conducted using TUNEL detection kits according to the manufacturer's instruction (TdT-FragEL^TM^ DNA fragmentation detection kit, Cat. No. QIA33, Calbiochem, Germany). The sections were deparaffinized and hydrated using graded alcohols and then permeabilized with a proteinase K (2 mg/ml, 1:100 in 10 mM Tris, pH  = 8) solution for 20 minutes at room temperature. The endogenous peroxidase was inactivated by 30% H_2_O_2_ (1:10 in methanol) for 5 minutes at room temperature and then washed with TBS (20 mM Tris pH  = 7.6, 140 mM NaCl). The sections were incubated with TdT buffer at room temperature for 20 minutes followed by incubation with a TdT labeling reaction mixture in a humid atmosphere at 37°C for 90 minutes. The reaction was terminated with a stop buffer. The sections were then incubated at room temperature for 30 minutes with a streptavidin – horseradish peroxidase conjugate. The signals were visualized using 3,3′-diaminobenzidine tetrahydrochloride. The sections were then counterstained with hematoxylin, and the nuclei of the positive cells were stained dark brown. Eight high-power visual fields (×400) were selected in each section to calculate the apoptotic index [AI  =  (number of TUNEL-positive cells)/(total number of the nucleated cells)].

### Statistical analysis

All data were presented as the mean ± SD. One-way analysis of variance (ANOVA) was used to compare the Ngb mRNA levels, Ngb protein levels, MDA levels, and AIs among the groups. The BBB scale data were analyzed with repeated-measures ANOVA. A P-value of less than 0.05 was considered significant. All statistical analyses were performed using SPSS 16.0 (SPSS Inc., IL, USA).

## Results

### Culture and characteristics of BMSCs

**Figure 1:**
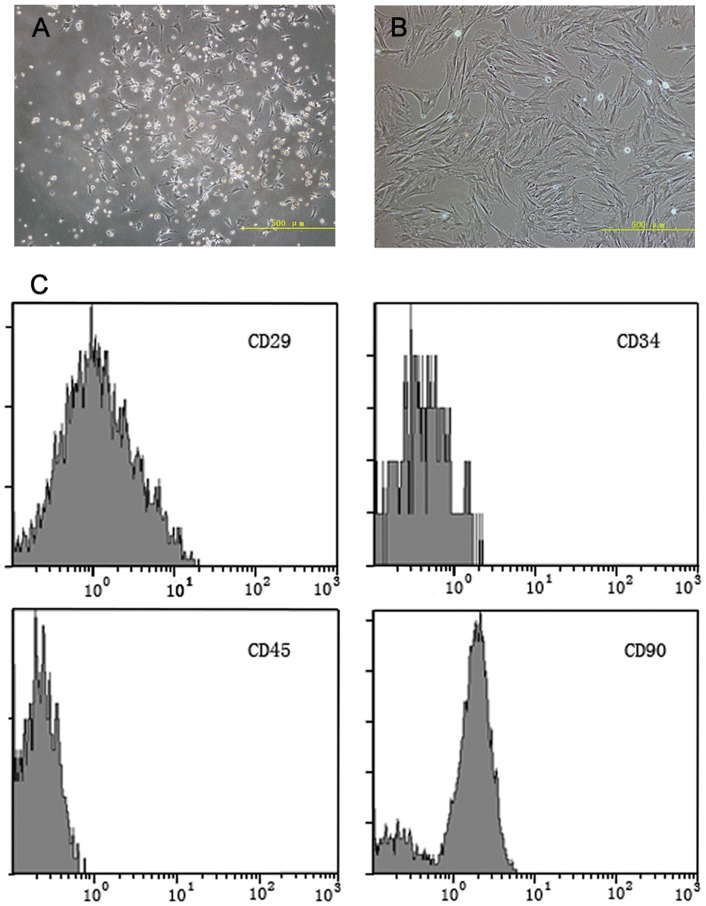
Morphology and phenotypic characterization of cultured BMSCs. (A) Primary BMSCs at 5 days of culture (magnification: ×100). (B) BMSCs at passage 3 (magnification: ×100). (C) Flow cytometric analysis of cultured BMSCs with CD29, CD34, CD45, and CD90 antibodies. [Bar  = 500 µm (A, B)].

BMSCs cultured as plastic adherent cells were maintained *in vitro*. The morphological features of the BMSCs are shown in Figs. 1A and 1B. Characteristic flattened and spindle-shaped cells can be recognized.

BMSCs possess a number of markers [31], [32]. To characterize the phenotype of the BMSCs, we detected cell markers such as CD29, CD34, CD45, and CD90 by flow cytometric analysis (Fig. 1C). The third passage of BMSCs were found to express CD29 and CD90 but not CD34 or CD45.

### Transferred gene expression *in vitro*

**Figure 2:**
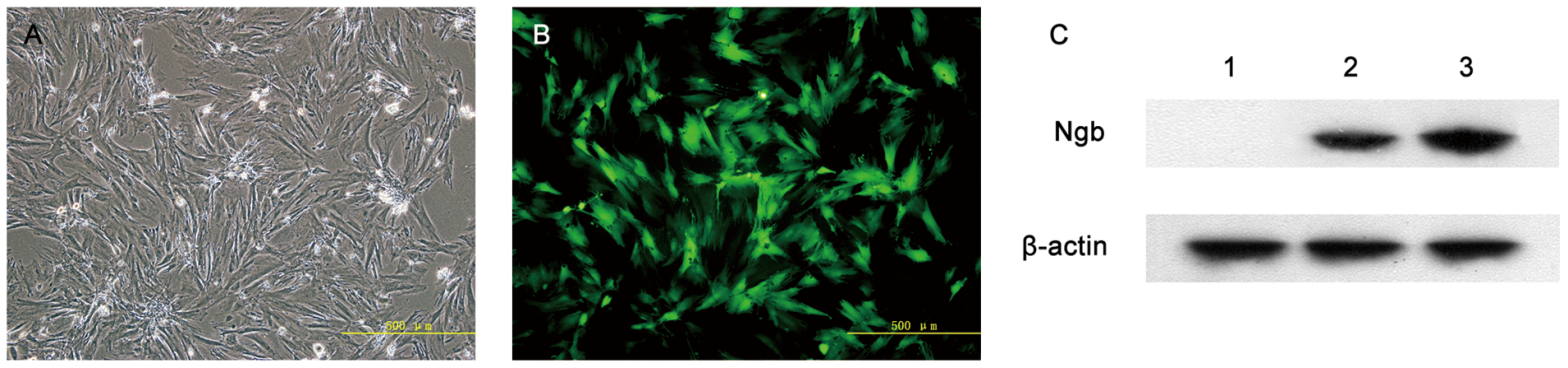
Transduction of Ngb into BMSCs with LV-Ngb. (A, B) eGFP expression in BMSCs 6 days after Ngb – eGFP transduction (magnification: ×100). (A) Phase contrast. (B) Fluorescence microscopy. (C) Western blot detection of Ngb protein expression (*lane 1*, before transduction; *lane 2*, 3 days after transduction; *lane 3*, 6 days after transduction) [Bar  = 500 µm (A, B)].

First, green fluorescence was identified in the cytoplasm of BMSCs 48 hours after infection with a fluorescence microscope, and the eGFP expression reached its peak 6 days following SCI (Fig. 2B). More than 80% of the LV-Ngb-infected BMSCs could be detected, indicating high transduction efficiency of LV-Ngb to BMSCs in this study.

We subsequently examined the levels of transferred gene expression of Ngb *in vitro* by Western blot analysis (Fig. 2C). The conditioned medium from Ngb-BMSC cells contained high levels of Ngb, whereas no detectable level of Ngb was observed in the medium from the plates with uninfected BMSCs.

### Detection of fluorescence in injured spinal cords

**Figure 3:**
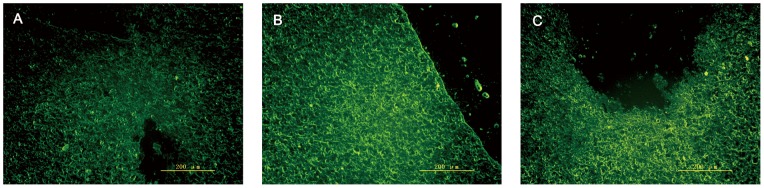
Fluorescence microscopy of the injured spinal cords. eGFP fluorescence detected with a fluorescence microscope on days 7 (A), 14 (B), and 21 (C) after SCI (magnification, ×200) [Bar  = 200 µm (A, B,C)].

On days 3, 7, 14, and 21 following SCI, the injured spinal cord tissues were removed, and frozen sections were prepared. eGFP fluorescence was observed clearly under a fluorescence microscope (Fig. 3).

### Overexpression of Ngb gene and protein in injured spinal cords after Ngb-BMSC treatment

**Figure 4:**
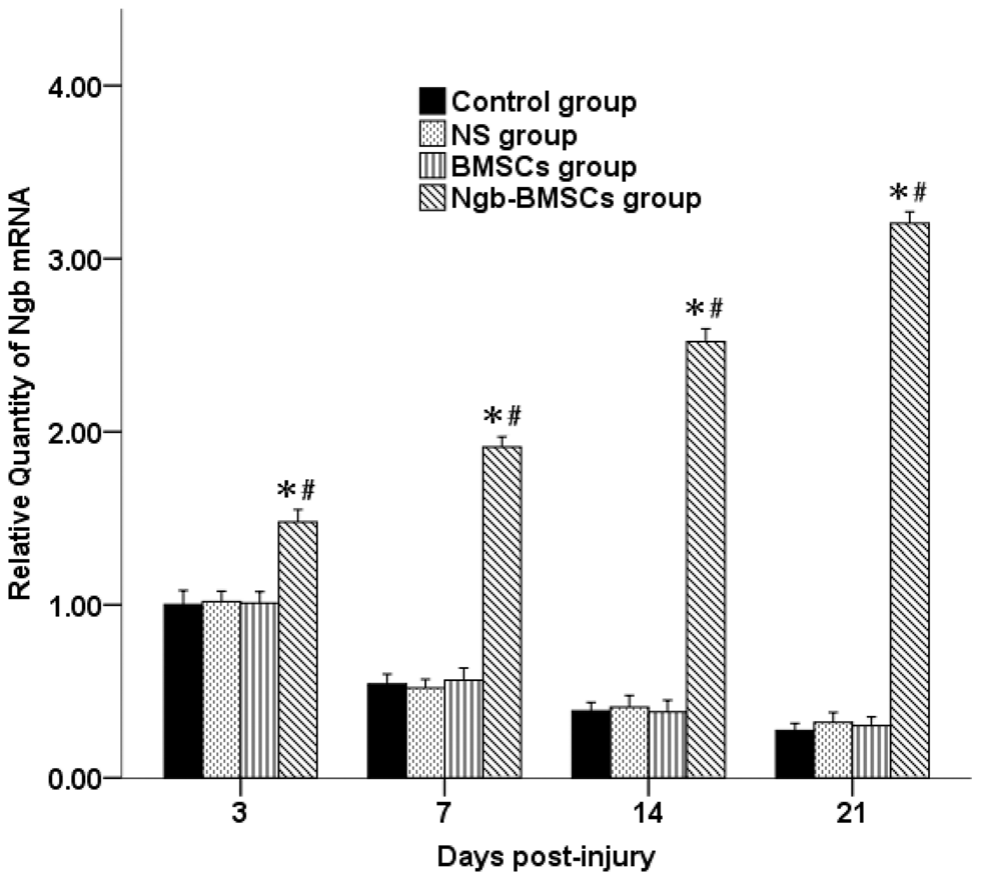
Ngb mRNA expression in the injured spinal cords on days 3, 7, 14, and 21 after SCI in each group. The data were plotted as mean ± SD (n = 6). ^*^ *P*<0.05 versus the Control and the NS groups (one-way ANOVA), ^#^ *P*<0.05 versus the BMSC group (one-way ANOVA).

As shown in Figure 4, the expression of the Ngb gene on days 3, 7, 14, and 21 following SCI in the spinal cords dissected from the rabbits in all four groups were assessed by qRT-PCR. In the Control group, the level of Ngb mRNA expression began to increase 3 days following SCI and then gradually decreased on days 7, 14, and 21. No difference was observed in the Ngb mRNA expression among the Control, NS, and BMSC groups. However, the level of Ngb mRNA expression in the Ngb-BMSC group was significantly higher on days 3, 7, 14, and 21 compared with the expression in the Control, NS, and BMSC groups (*P*<0.05).

**Figure 5:**
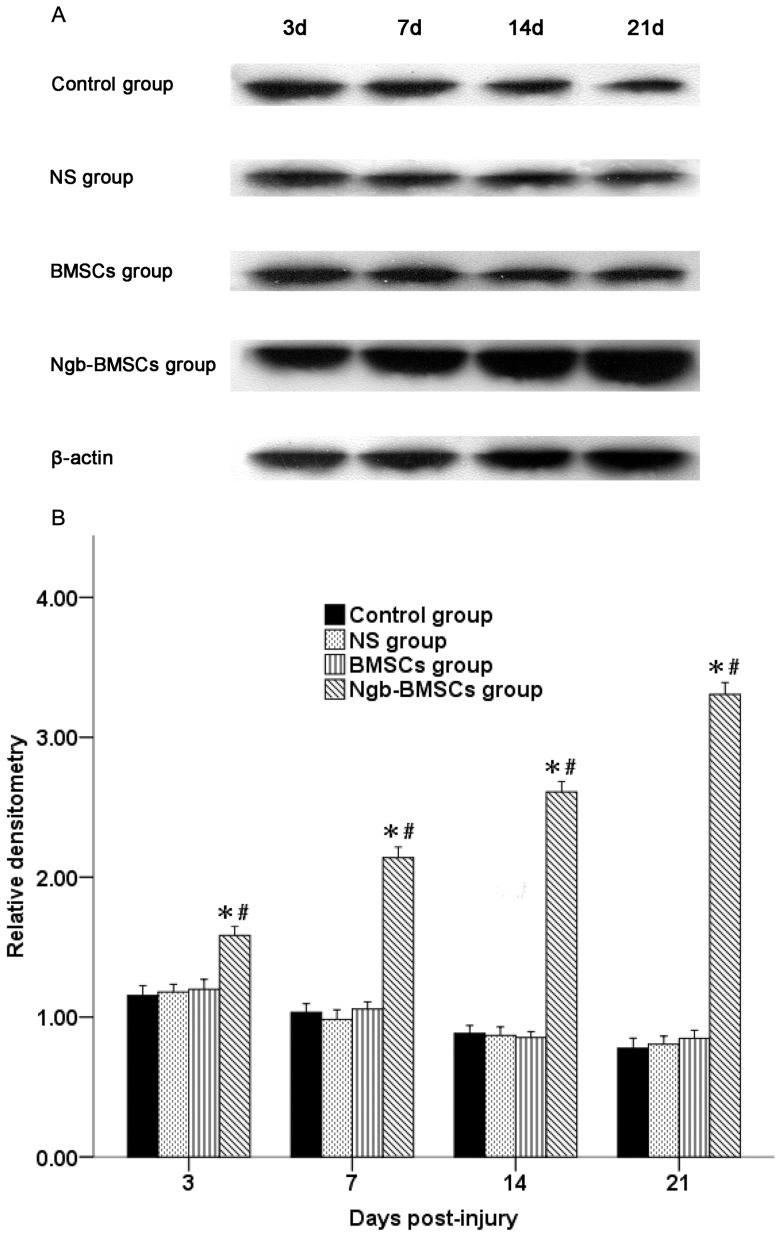
Detection of Ngb protein in the spinal cords. (A) Ngb and β-actin detected by Western blot in the spinal cord lysates obtained from four groups at four different time points. The blots are representative examples from six experiments per group per time point. (B) The quantitative mean ± SD data shows the level of Ngb protein expression. The band density is normalized as the ratio of Ngb: β-actin (n = 6). ^*^ *P*<0.05 versus the Control and the NS groups (one-way ANOVA), ^*^ *P*<0.05 versus the BMSC group (one-way ANOVA).

As shown in Figure 5, the Ngb protein expression was investigated by Western blot analysis. In the Control group, the level of Ngb protein expression first showed a temporary increase and then gradually decreased on days 7, 14, and day 21. No significant difference was observed in the Ngb protein expression among the Control, NS, and BMSC groups. However, the Ngb protein expression in the Ngb-BMSC group increased more significantly on days 3, 7, 14, and 21 compared with the levels in the Control, NS, and BMSC groups (*P*<0.05).

### Neurological outcome

**Figure 6:**
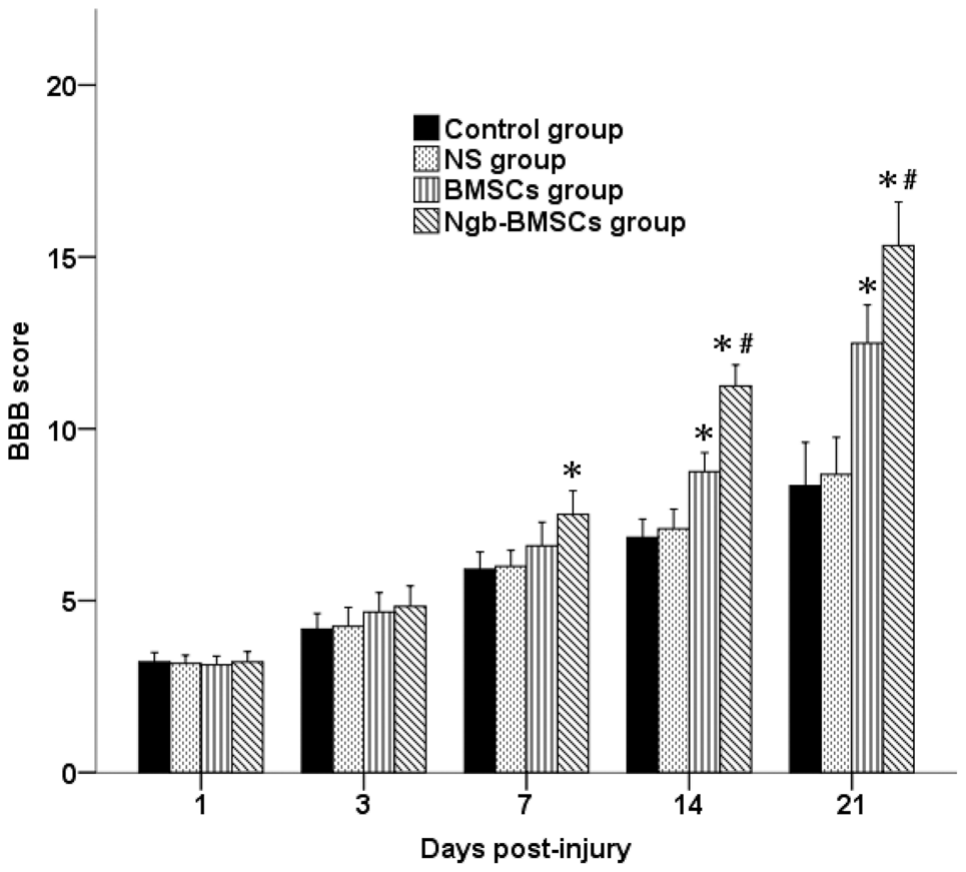
Hindlimb functional assessment with the BBB rating scale on days 1, 3, 7, 14, and 21 after SCI in each group. The data are plotted as mean ± SD. ^*^ *P*<0.05 versus the Control and the NS groups (repeated-measures ANOVA), ^*^ *P*<0.05 versus the BMSC group (repeated-measures ANOVA).

The evaluations of the hindlimb locomotor functions in each group on days 1, 3, 7, 14, and 21 following SCI are presented in Figure 6. All rabbits became paraparetic, and no significant difference was observed among the four groups 1 day after SCI. Partial improvements were observed, and no significant difference was indicated among the four groups 3 days after SCI. However, the Ngb-BMSC group obtained significantly higher BBB scores than did the Control and the NS groups 7 days after SCI (*P*<0.05). On days 14 and 21 after SCI, the BBB scores in the BMSC and Ngb-BMSC groups were significantly higher than those in the Control and the NS groups (*P*<0.05), whereas the BBB scores in the Ngb-BMSC group were significantly higher than those in the BMSC group (*P*<0.05).

### Changes in MDA levels in injured spinal cords

**Figure 7:**
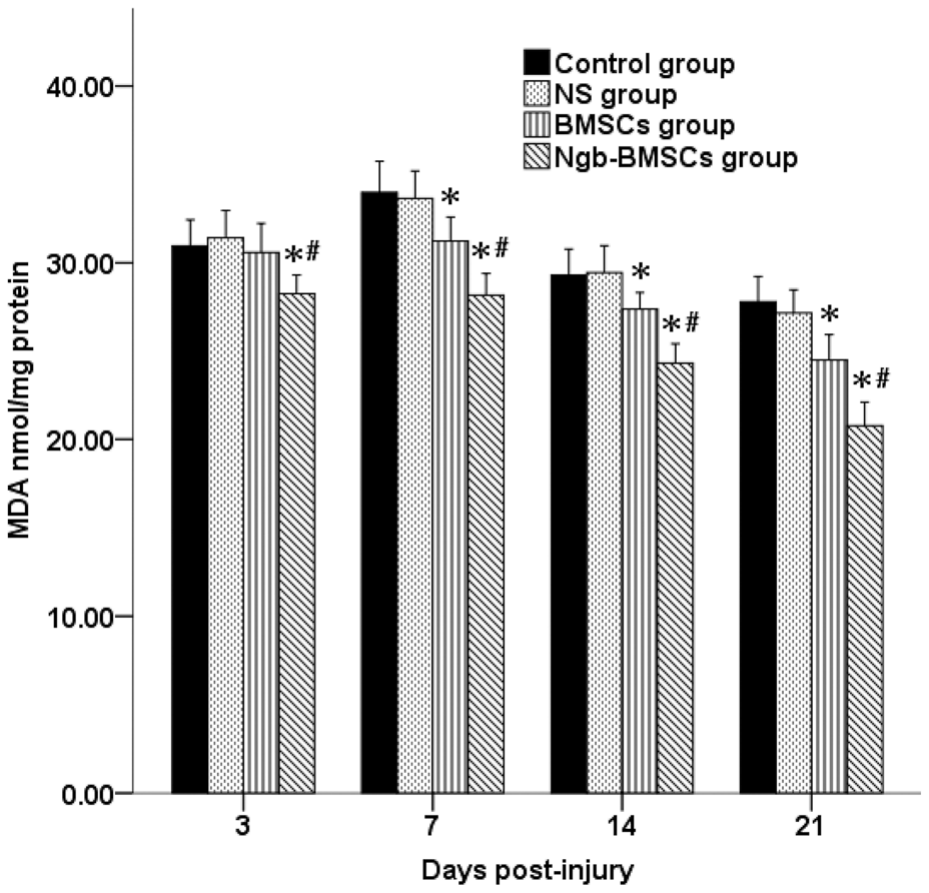
MDA levels in the injured spinal cords in the four groups at four different time points. Data were plotted as mean ± SD (n = 6). ^*^ *P*<0.05 versus the Control and the NS groups (one-way ANOVA), ^#^ *P*<0.05 versus the BMSC group (one-way ANOVA).

The MDA levels in the injured spinal cords are shown in Figure 7. The MDA levels in the four groups increased 3 days after SCI. Those in the Ngb-BMSC group were significantly lower compared with those in the Control, NS, and BMSC groups (*P*<0.05). However, no significant difference was observed among the Control, NS, and BMSC groups. On days 7, 14, and 21 following SCI, the MDA levels in the BMSCs and Ngb-BMSC groups were significantly lower compared with those in the Control and the NS groups (*P*<0.05). The MDA levels in the Ngb-BMSC group were significantly lower compared with those in the BMSC group (*P*<0.05).

### TUNEL labeling

**Figure 8:**
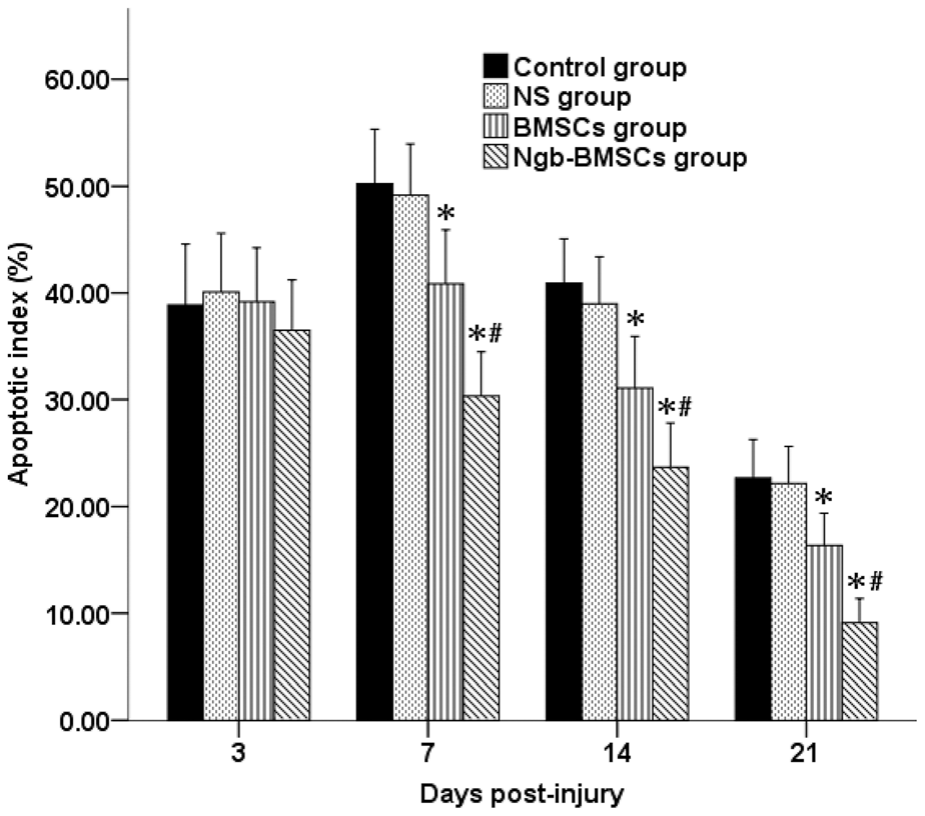
Apoptotic indexes at four different time points in each group. The data are plotted as mean ± SD (n = 6). ^*^ *P*<0.05 versus the Control and the NS groups (one-way ANOVA), ^#^ *P*<0.05 versus the BMSC group (one-way ANOVA).

**Figure 9:**
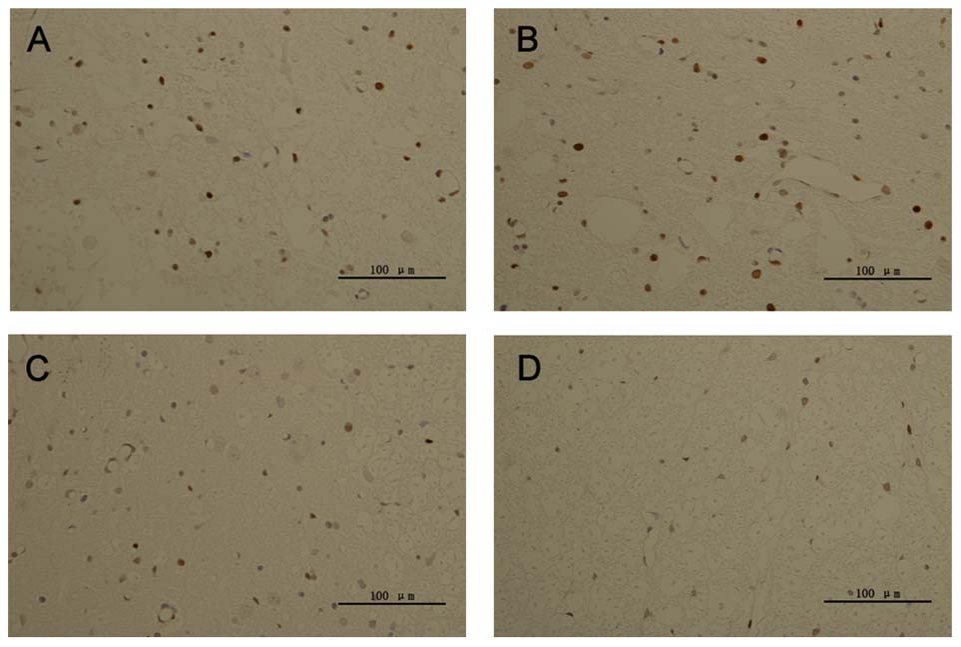
Apoptosis in the injured spinal cords on day 7 after SCI (magnification, ×400). (A) Control; (B) NS; (C) BMSC; (D) Ngb-BMSC groups. The TUNEL positive cells were stained dark brown. The number of positive cells was significantly lower in the Ngb-BMSC group compared with the numbers in the Control, NS, and BMSC groups [n = 6, Bar  = 100 µm (A, B, C, D)].

The AIs at four different time points post-SCI in the four groups are shown in Figs. 8 and 9. A small number of TUNEL-positive cells were detected 3 days after SCI, and no significant difference was observed among the AIs in the four groups. On days 7, 14, and 21 following SCI, the AIs in the BMSC and Ngb-BMSC groups were significantly lower compared with the AIs in the Control and the NS groups (*P*<0.05), and the AIs in the Ngb-BMSC group were significantly lower compared with the AIs in the BMSC group (*P*<0.05).

## Discussion

The development of a therapy that can reduce the evolution of secondary damage in injured spinal cords is important. The damage caused by secondary injury is more severe than that caused by primary injury. Secondary injury is a process of initiative modulation at the cellular and molecular levels, which is reversible and controllable [33]. Recent studies have focused on the effects of cell-based gene therapy on secondary injury [34], [35]. In the present study, we have shown that BMSCs can be efficiently transduced with lentivirus vectors. Ngb overexpression can also be achieved in the injured spinal cord by Ngb gene-modified BMSC transplantation. We have also found that Ngb gene-modified BMSCs can improve the protective effect of BMSC therapy for SCI. To the best of our knowledge, the present study is the first to demonstrate the effects of Ngb gene-modified BMSCs on traumatic SCI.

BMSCs are easy to harvest, culture, and expand *in vitro* and have been identified to exhibit low immunogenicity [36]–[39]. BMSC transplantation was observed to significantly improve the hindlimb locomotor function in the SCI model [14], [40], [41]. BMSCs can act as cellular vehicles for gene delivery and are being developed to treat SCI [41], [42]. Therefore, BMSCs are likely candidates as ideal delivery vectors in gene therapy for SCI. The morphological features of the BMSCs in the present study were similar to those of previously reported BMSCs [43], [44]. The results of flow cytometric analysis showed that our isolated BMSCs expressed BMSC markers (CD29, CD90) instead of hematopoietic lineage markers (CD34, CD45). These results are consistent with those of previous studies [45], [46].

Ngb is a monomeric globin (molecular mass ≈17 kDa) discovered by Burmester *et al*. [15] in 2000. Previous studies have suggested that Ngb can function as an important neuroprotective molecule [20], [21], [47], [48] induced by neural hypoxia and cerebral ischemia. Ngb protects neurons from hypoxic and ischemic injuries [18], [19], [20], [49]. A recent study has indicated that Ngb overexpression in the brain helps reduce the infarct following ischemia [18], [19]. Ngb has also been found to protect neurons against damaging ROS [50] and Alzheimer's disease [51].

The transfer of specific genes for therapeutic purposes provides a valuable approach to treating SCI [52], [53]. Recombinant lentiviral vectors have been proven superior to other vectors in terms of gene transfer because the former can provide long-term expression of the therapeutic gene as well as efficiently transduce nondividing cells, including neurons [54]. BMSCs have recently been genetically enhanced by lentiviral vectors to stabilize gene expression and increase their therapeutic potential [55], [56]. Therefore, in the present study, we used a lentiviral vector to deliver Ngb to the BMSCs *in vitro*. We found that LV-Ngb had high transductive efficiency to BMSCs, and Ngb gene-modified BMSCs contained a high level of Ngb protein. We also injected Ngb gene-modified BMSCs into the injured spinal cords of the rabbits *in vivo* and found that Ngb overexpression in the injured spinal cords was achieved successfully.

The BBB open field scoring system is frequently used for the quantitative evaluation of the neurological status after SCI in animals. Using the BBB scale, we demonstrated a progressive recovery over time in all four groups. The functional recovery in the Ngb-BMSC and BMSC groups showed a more statistically significant improvement compared with the recoveries in the control and the NS groups. Significant functional improvements in hindlimb movements were also observed in the Ngb-BMSC group compared with those in the BMSCs group. These results indicate that Ngb overexpression by Ngb gene-modified BMSCs can improve the functional recovery of BMSCs in the SCI rabbits.

Despite our demonstration of the neuroprotective effect of Ngb overexpression, the neuroprotective mechanisms of the Ngb overexpression have not been fully elucidated [49], [57]–[59]. One possible mechanism is that Ngb functions as a ROS regulator [21], [23]. ROS-induced lipid peroxidation is one of the most important precipitating components of neuronal degeneration after SCI. MDA is widely known as an important indicator for lipid peroxidation. MDA levels in animals exposed to traumatic SCI can significantly increase [60]–[62]. Previous experimental studies have also reported that Ngb overexpression is neuroprotective against direct oxidative stress [63], [64]. The reduction in the MDA level in Ngb-Tg mouse brains suggests that Ngb can promote neuronal survival by reducing oxidative stress after focal cerebral ischemia [65]. The MDA levels in the present study increased significantly in the injured spinal cords, and the Ngb overexpression significantly attenuated the increase in MDA levels in the Ngb-BMSC group compared with the levels in the Control, NS, and BMSC groups. This result may be attributed to its ability to eliminate ROS. The protective effect of the Ngb overexpression in the SCI may be caused by the reduction in the lipid peroxidation that occurred in the neurons of the spinal cords.

Another possible mechanism is that Ngb can protect neuronal cells from apoptosis [66]. Ngb proteins exhibit high concentrations (up to 100 µM) in neurons and the retina [67], and it is closely associated with the mitochondria [67]. The induction of mitochondrial dysfunction can lead to overproduction of superoxide anions, which is essential to promote enhanced apoptosis in neuronal cells [68]. Thus, maintaining the mitochondrial function is crucial for cell survival. Ngb can possibly reduce a small amount of cytochrome c leaking from the damaged mitochondria, thereby inhibiting the intrinsic apoptosis pathway [69]. Apoptosis is an important mediator of the secondary damages after SCI [70], [71]. In the present study, we examined the expression of the apoptotic cells in the traumatic spinal cords by using the TUNEL detection kits. The number of apoptotic cells in the spinal cords significantly increased after SCI. Ngb overexpression also significantly reduced the expression of apoptotic cells in the Ngb-BMSC group compared with the reductions in the Control, NS, and BMSCs groups. This result suggests that Ngb overexpression has a role in preventing apoptosis in traumatic SCI.

However, several limitations are included in the present study. First, only a single dose of the BMSC or Ngb-BMSC treatment was used based on the previous findings. Second, immunofluorescence histochemistry for Ngb in the injured spinal cord tissues after injection of Ngb gene-modified BMSC was also needed. Third, the number of animals in each group at each time point was small. Fourth, no other objective measurement was performed to evaluate the hindlimb locomotor functions aside from the BBB rating scale. Finally, only one pathological section was extracted at the T9 level from each rabbit. Therefore, these issues should be addressed in future studies.

To summarize, the present study shows that Ngb overexpression may improve lipid peroxidation and prevent apoptosis after traumatic SCI. Ngb gene-modified BMSCs can strengthen the therapeutic benefits of BMSCs in reducing secondary damage and improving neurological outcome after traumatic SCI. Therefore, the combined strategy of BMSC transplantation with Ngb gene therapy exhibits potential for the treatment of traumatic SCI.
